# Brazilian version of the OHIP 14 Periodontal Disease Questionnaire: cross cultural adaptation and validation

**DOI:** 10.1590/1807-3107bor-2025.vol39.002

**Published:** 2025-01-20

**Authors:** Timilly Mayra MARTINS-CRUZ, Kaio Henrique SOARES, Juliana Helena Gomes LEAL, Olga Dumont FLECHA, Dhelfeson Willya DOUGLAS-DE-OLIVEIRA, Patrícia Furtado GONÇALVES

**Affiliations:** (a) Universidade Federal dos Vales do Jequitinhonha e Mucuri UFVJM, School of Biological and Health Sciences, Department of Dentistry, Diamantina, MG, Brazil.; (b) Universidade Federal dos Vales do Jequitinhonha e Mucuri – UFVJM/MG, Faculdade de Letras, Department, Diamantina, MG, Brazil.

**Keywords:** Periodontal disease, Validation study, Quality of life

## Abstract

Although it is recognized that periodontal disease negatively impacts quality of life, there is no validated instrument to assess this impact in Brazil. This study aimed to translate, cross-culturally adapt, and validate the OHIP 14 PD (Oral Health Impact Profile Applied to Periodontal Diseases) for application among Brazilian patients. The original instrument was translated and validated into Brazilian Portuguese in a cross-sectional study with 110 participants recruited from a Dental School clinic. The sample was divided into two groups: 55 with periodontal disease and 55 without periodontal disease. The instrument was self-administered twice within an interval of 7 to 10 days for patients with periodontal disease. The psychometric properties of the Brazilian version were verified using internal consistency (Cronbach’s α) and the reliability through the test-retest method (ICC, intraclass correlation coefficient), convergent validity (Spearman correlation), and discriminant validity (Mann-Whitney test), with p <0.05. Most of the sample consisted of women (n=69; ±40.65 years). The OHIP 14 DP - Br showed excellent internal consistency (α=0.997) and outstanding reliability using the test-retest method (ICC=0.945, p<0.001). There was a significant correlation between the scores obtained in all seven domains between this questionnaire and self-perceived gingival health (p=0.023). This study provides psychometric evidence supporting the cross-cultural validity of the OHIP 14 DP - Br version for use in Brazil.

## Introduction

The clinical parameters used to assess oral diseases like dental caries or periodontal diseases fall short of capturing the comprehensive idea of health defined by the World Health Organization (WHO), especially concerning mental and social well-being.^1,2^ This gap has spurred the need for new health assessment tools that prioritize patient-reported outcomes, known as PROMs (Patient-Reported Outcome Measures), diverging from traditional clinical evaluations of disease status.^2^

Consequently, researchers have developed standardized and alternative measures, typically validated questionnaires, to gauge oral conditions’ physical, psychological, and social impact on an individual’s life.^1^ Utilizing PROMs allows clinicians to directly gather information on health outcomes from patients, facilitating a safer and more accurate assessment of changes in their condition over time.^2,3^

Periodontal disease is considered one of the most common oral conditions in the human population,^4^ and studies suggest that it can negatively impact the oral-health-related quality of life.^5,6^ The disease encompasses various chronic and pathological inflammatory conditions of the gingiva, alveolar bone, and periodontal ligament. Such conditions can lead to the collapse of the tooth’s supporting tissues, triggering its progressive destruction, bone resorption, and, eventually, tooth loss.^7-9^

Many instruments have been developed to assess the impact of oral diseases on people’s daily lives, thus affecting their oral-health-related quality of life.^5,6^ Among these instruments, the OHIP 49 (Oral Health Impact Profile)^10^ and its reduced version - the OHIP 14 - published in 1997^10^ are primarily used based on Locker’s biopsychosocial model.^10^ These questionnaires were validated and translated into several languages, allowing their scores to be compared in different populations.^11^

Given the heterogeneity of generic instruments currently used to assess periodontal patients’ self-reported quality of life, developing a specific tool related to periodontal disease is strongly recommended, since this can increase reliability and validity of patient-reported outcome measures.^3^ Thus, an instrument named Oral Health Impact Profile Applied to Periodontal Diseases (OHIP 14 PD), originally in Spanish, was developed and validated in Mexico to assess periodontal disease based on the original OHIP questionnaire specifically.^12^

The cross-cultural adaptation and validation of an instrument in other languages allow for existing data to be compared across cultures and countries, facilitating communication and information exchange within the scientific community. Introducing a new questionnaire about the quality of life in a country with a different language or culture from its origin necessitates systematic validation.^13^ Currently, the translated and validated Brazilian version of this condition specific instrument (OHIP 14 PD) is unavailable.

Considering the sociocultural reality in Brazil and the latest epidemiological findings on periodontal diseases in this country,^14^ this study aimed to translate, cross-culturally adapt, and validate the original version of the OHIP 14 PD^12^ for the population of adult Brazilian periodontal patients. The adapted version in Portuguese, pointing out valid psychometric variables (construct validity and reliability), may significantly impact scientific research, therapeutic approaches, and intervention plans for health promotion and prevention. Clinicians should be able to monitor changes in the aspect of health over time and complement clinical data. Moreover, this PROM tool can be a valuable instrument to capture the self-perceived health status of periodontal patients in Brazil.

## Methodology

### Ethical principles

This study was approved by the Research Ethics Committee of the Federal University of Jequitinhonha e Mucuri Valleys under protocol number 49276821.4.0000.5108. All participants were informed about the purpose of this study and signed the Free and Informed Consent Form following resolution 466 of the National Health Council of 12/12/2012.

### Description of OHIP 14 PD

The OHIP 14 PD^12^ was initially developed in Spanish (Mexico) in 2017, adapting the questions from the OHIP 14 (based on the OHIP 14 in its Spanish version 14)^15^ to assess periodontal conditions. Its content was validated and showed very high internal consistency, using analysis techniques appropriate to the ordinal nature of the variables and a sample with an adequate size.^16^ It resulted in a specific instrument for assessing periodontal disease’s physical, social, and psychosocial impact in adults, with its items considered sufficient, coherent, relevant, and clear in writing.^16^

Thus, the actual OHIP 14 PD,^12^ a condition-specific instrument, consists of 14 items in 7 domains/dimensions (functional limitation, physical pain, psychological discomfort, physical disability, psychological disability, social disability, and social disadvantage). It is answered on a 5-point scale, ranging from 0 to 4, where 0 corresponds to “Never” to 4 being “Almost always”. The final score is calculated by adding the scores of the 14 items, ranging from 0 to 56, according to a Likert scale^17^. Higher scores indicate a more significant negative impact of periodontal disease on quality of life.^12^

### Eligibility criteria

Participants over 18 years of age, who had at least 16 teeth, excluding third molars, who understood written and verbal commands, and who had the PSR exam (Periodontal Screening and Recording)^18^ in their medical records in the last six months were selected, both for the the pre-test and test phase. Thus, participants with the following criteria were included in this study: a) Patients without periodontal disease (PSR score ≤ 2; n=55) and b) Patients with periodontal disease (PSR score ≥ 3; n = 55).

However, individuals undergoing active periodontal or orthodontic treatment, those wearing complete dentures, or individuals who declined to participate were excluded from the study. Additionally, failure to answer at least one question from the OHIP 14 DP - Br questionnaire served as an elimination criterion, ensuring data integraty and participant compliance throughout the research process.

### Development of the Brazilian version of OHIP 14 PD12

This study was designed according to the COnsensus-based Standards for the selection of health Measurement INstruments (COSMIN) recommendations for patient-reported outcome measurement instruments.^19^ The translation and cross-cultural adaptation process was conducted by a group of experienced researchers, following the protocol by Beaton et al. ^20^ (Figure) and the COSMIN guidelines,^19^ after contacting the original authors to obtain further information and clarify possible doubts.

**Figure:**
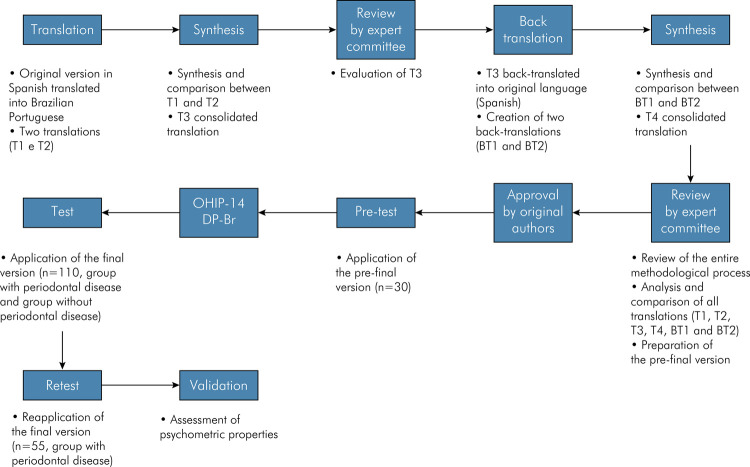
Flowchart of the translation, cross-cultural adaptation, and validation of the OHIP 14 PD instrument, based on the protocol by Beaton et al.20 and prepared by the authors.

Initially, the conceptual translation of the questionnaire from Spanish to Brazilian Portuguese was carried out by two independent Brazilian translators, native Brazilian Portuguese speakers and fluent in Spanish, one a dentist and the other not.

These two versions (T1 and T2) were then synthesized (T3) based on the available versions, compared, and then abbreviated. The translated version (T3) was presented for appreciation by the expert committee, and soon after, the Spanish-to-Portuguese version was conducted for back-translation. This committee of experts was composed of two Professors of Periodontics, one Professor of Dental Materials with experience in validating questionnaires, one Professor of Spanish Language, and one Ph.D. candidate and aimed to consolidate and evaluate, after consensus, all versions of the questionnaire and develop the pre-final version for field testing.

The back-translation process involved two independent back-translators native to Spanish- speaking countries and fluent in Brazilian Portuguese, one a dentist and the other not. Again, the back-translated versions (BT1 and BT2) were synthesized (T4) and presented to the expert committee to verify this version’s semantic, idiomatic, cultural, and conceptual equivalence. After the assessment of all translations (T1, T2, T3, BT1, BT2, and T4), they were checked in terms of clarity, understanding, and adequacy of the objectives of the questionnaire. There were no differences between the versions. The authors of the original version were again contacted for appreciation of the produced version (T4) and its evaluation. They did not suggest any changes. In this way, the pre-final version was established.

Subsequently, the pre-final version (pre-test) of the Brazilian Portuguese version of the instrument was applied to a convenience sample composed of 30 individuals who attended the dental clinics of the Department of Dentistry at the Federal University of Jequitinhonha e Mucuri Valleys (UFVJM), located in Diamantina – MG, in the southeastern region of Brazil. In addition to clinic patients, employees and students were invited, all selected according to the eligibility criteria for this study. These individuals did not participate in the assessment tests of the instrument’s psychometric properties and did not compose the final sample. After self-reporting the items, each participant was asked about difficulties understanding or answering instructions. Again, there were no participants’ suggestions or doubts.

At this stage, the instrument was renamed OHIP 14 DP - Br and proceeded to the test phase and subsequent statistical analysis of its psychometric properties.

### Evaluation of the psychometric properties of the OHIP 14 DP - Br

The reliability and validity process followed the COSMIN checklist.^19^ In order to evaluate the psychometric properties of the OHIP 14 DP- Br, the questionnaire was self-administered (paper based) by volunteers attending the dental clinics at UFVJM. The sample size calculation was based on an alpha coefficient of 0.5, a beta coefficient of 0.10, and a correlation coefficient 0.05. Thus, 100 participating individuals were estimated to validate the questionnaire, with 110 participants as the minimum sample for this study.

The data collection took place from January to May 2022. The questionnaires were administered to patients attending the UFVJM dental clinics, who had been registered and clinically evaluated within the previous six months, and who met all eligibility criteria.

Clear and concise instructions were given to all participants about self-completion of the questionnaire, with no questions being allowed to the researcher during the responses. The questionnaire consists of 14 questions, with responses recorded according to the Likert Scale,^17^ where 0c = Never; 1c = Almost never; 2c = Occasionally; 3c = Frequently; and 4c = Very often.

After 7 to 10 days from the first application, only the participants of the group of patients with periodontal disease (n = 55) were invited to return to the university and answer the questions again, thus configuring the test-retest method.^21^

Only individuals with periodontal disease were asked how they classified their gingival health to assess convergent validity on a scale from 0 to 10.

### Statistical analysis

The results were submitted to descriptive statistical analysis (means and standard deviations). Calculations were performed using the SPSS program (Statistical Package for Social Sciences®, Version 22.0, Chicago, USA).

The reproducibility stability of the questionnaire (test-retest) was calculated using the intraclass correlation coefficient (ICC) among the 55 participants with periodontal disease who answered the questionnaire. This index was calculated based on single-measure consistency under the mixed two-way model. The ICC is considered to have poor agreement when the value is ≤ 0.40, moderate agreement when 0.41 to 0.60, good agreement when 0.61 to 0.80, and for excellent agreement, the ICC must be > 0.81.^22^

The reliability and internal validity of the adapted questionnaire were evaluated by calculating Cronbach’s Alpha for each item and each dimension. An alpha value higher than 0.70 was considered acceptable, presenting a satisfactory internal consistency.^23^

Convergent validity was tested using the correlation test (Spearman correlation) between the OHIP 14 DP - Br scores and the gingival health classification. The underlying hypothesis was that patients who rate their oral status as deficient would score higher on the OHIP 14 DP - Br. Correlation coefficients were considered to indicate poor correlation when < 0.20, low when 0.21 – 0.40, fair when 0.41 – 0.60, very good correlation when 0.61 – 0.80, and to indicate excellent correlation when > 0.8.1^22^ Correlations between variables were expected to be very good or negative.

Discriminative validity was tested using the Mann-Whitney test, comparing the mean scores on each subscale and total OHIP 14 DP - Br between participants with and without periodontal disease. The hypothesis was that participants with periodontal disease had higher scores on the OHIP 14 DP - Br, indicating a worse oral health-related quality of life (OHQoL).

## Results

### Adaptation to the Brazilian Portuguese version

Assessments were conducted by a panel of experts demonstrating semantic equivalence between the original version and the two translations. Semantic equivalence was evaluated based on the comparison between the back-translated versions and the original questionnaire. The expert panel suggested that items begin with the treatment pronoun “you”. In addition, some Spanish expressions were altered to understand Brazilian Portuguese better. Regarding the scale of responses, the panel of experts made the following substitutions: “Casi nunca” for “Rarely”; “Ocasionalmente” for “Few times”; “Frecuentemente” for “Sometimes” and “Muy frecuentemente” for “Almost always”. After that, the response scale was considered relevant and without inconsistencies.

### Participant characteristics during the validation study

The validation study was conducted with 110 participants who made up the final sample for the OHIP 14 DP - Br psychometric tests. It was divided equally into two groups, without periodontal disease (n = 55) and with periodontal disease (n = 55). The sample consisted mainly of women (62.7%, n = 69), and the mean age was 40.65 (± 13.54 years).

### Psychometric properties of OHIP 14 DP - Br

All data collection was completed without any deletions or complications. The response time was around 15 minutes. The final score of the seven dimensions, which could be scored from 0 to 56, presented the following average score for patients with periodontal disease: 30.77 (±1 4.39), and for patients without the disease, it demonstrated the average of 8.50 (± 9.50), as shown in Table 1. In all evaluated dimensions, including the total average, a statistically significant value (p < 0.001) was observed between the two evaluated groups.

**Table 1:** Association between OHIP 14 DP - Br scores and periodontal disease (Discriminant validity).

Variable	Periodontal disease		Periodontal disease		p-value
	present		absent		
Dimensions	Mean	SD	Mean	SD	
Functional limitation	4,43	2,46	1,16	1,59	< 0,001
Physical pain	4,24	2,4	1,92	1,95	< 0,001
Psychological discomfort	4,09	2,61	1,2	1,67	< 0,001
Physical disability	4,85	2,46	0,98	1,75	< 0,001
Psychological disability	5,36	3,02	1,43	2,6	< 0,001
Social disability	3,5	2,81	0,63	1,51	< 0,001
Handicap	4,45	2,35	1,16	1,84	< 0,001
Total score	30,77	14,39	8,5	9,5	< 0,001

Mann-Whitney’s Test; SD: Standard deviation

The convergent validity was evaluated by correlating the scores obtained in each dimension of the OHIP 14 DP - Br of patients with periodontal disease and the self-classification of these participants regarding their gingival health status. The results (Table 2) indicated a negative and poor correlation for all instrument dimensions.

**Table 2:** Correlation between the OHIP 14 DP - Br and the global self-assessment of gingival health of participants with periodontal disease (Convergent validity).

Dimensions	Global self-assessment of gingival health	
	r_s_	p-value
Functional limitation	-0,265	0,046
Physical pain	-0,167	0,227
Psychological discomfort	-0,176	0,199
Physical disability	-0,311	0,021
Psychological disability	-0,347	0,009
Social disability	-0,334	0,013
Handicap	-0,163	0,234
Total score	-0,31	0,023

r_s_: Spearman correlation coefficient

Reliability was calculated according to participants’ responses with periodontal disease (n = 55) since it is a condition-specific instrument. The OHIP 14 DP - Br questionnaire showed excellent reliability results, both for internal consistency (Cronbach’s alph a = 0.945) and for the reliability of the test-retest method (ICC = 0.997; 95%CI: 0.995 – 0.998), evaluating all seven dimensions (Table 3). In this way, the ICC was considered an excellent agreement, while the internal consistency was considered acceptable for most of the evaluated dimensions.

**Table 3:** Mean score, test-retest reliability, and internal consistency of the OHIP 14 DP - Br dimensions and total score.

Dimensions	Mean	SD	ICC	95%CI (LE-UE)	p-value	Cronbach’s Alpha
Functional limitation	4,43	2,46	0,821	0,693 – 0,896	< 0,001	0,633
Physical pain	4,24	2,4	0,893	0,816 – 0,938	< 0,001	0,632
Psychological discomfort	4,09	2,61	0,852	0,746 – 0,913	< 0,001	0,815
Physical disability	4,85	2,46	0,833	0,713 – 0,903	< 0,001	0,772
Psychological disability	5,36	3,02	0,865	0,769 – 0,922	< 0,001	0,948
Social disability	3,5	2,81	0,859	0,759 – 0,918	< 0,001	0,799
Handicap	4,45	2,35	0,884	0,801 – 0,932	< 0,001	0,666
Total score	30,77	14,39	0,997	0,995 – 0,998	< 0,001	0,945

ICC: Intraclass correlation coeficiente; CI Confidence Interval; LE: Lower Extremity; UE: Upper Extremity

## Discussion

The OHIP 14 DP - Br version was the first to translate, cross-culturally adapt, and validate a specific self-administered instrument for assessing the impact of periodontal disease on the life of adults in Brazil.

A notable scientific appeal has been directed to the development of specific instruments, as these can present greater discriminant validity and responsiveness properties, such as greater sensitivity to the disease of interest, with the ability to detect particularities and certain aspects that can be confused or neglected in an evaluation with broader or more generic instruments.^24^

On the other hand, according to Fayers,^25^ it could be challenging to understand a phenomenon and validate the psychometric characteristics of this instrument, needing greater sensitivity and reflection in its interpretation^2^. However, in the present study, the psychometric assessments showed satisfactory construct validity and reliability for validation.

The OHIP 14 PD questionnaire, initially written in Spanish (Mexico), was systematically evaluated and presented content validity as an instrument used to analyze the impact of periodontal disease on the quality of life of affected individuals.^12,16^

Cross-cultural adaptation aims to ensure consistency in content and validity between the source and target versions of a questionnaire.^17^ The planning, structuring, and method of translation and cross-cultural adaptation used in the present study followed the protocol for cross-cultural adaptation processes of self-reported measures proposed by Beaton et al.^20^

**Table 4:** Internal consistency of the OHIP 14 DP - Br according to the items or questions (Q) of the instrument.

Items (question)	Scale mean if item deleted	Corrected item-total correlation	Cronbach’s Alpha if item deleted
Q1	28,39	0,334	0,918
Q2	28,8	0,696	0,906
Q3	29	0,563	0,911
Q4	28,31	0,592	0,91
Q5	28,91	0,625	0,908
Q6	28,52	0,669	0,907
Q7	28,35	0,644	0,908
Q8	28,35	0,709	0,905
Q9	28,06	0,761	0,903
Q10	28,19	0,731	0,904
Q11	28,94	0,672	0,907
Q12	29,15	0,637	0,908
Q13	29,26	0,621	0,908
Q14	27,89	0,498	0,913

Regarding the semantic, idiomatic, and conceptual equivalence of the proposed validation process, the authors of the original version and the panel of experts concluded that both the translations and the back-translations presented satisfactory outcomes concerning the original instrument without changing its purpose of use. It was verified that the protocol was systematically followed. According to the literature, this audit is intended to keep the content the same since a reasonable instrument translation has already been achieved at this point.^20^ Thus, the version for validation was obtained, called OHIP 14 DP - Br.

Some studies have questioned the validity of self-administered questionnaires about periodontal disease.^26,27^ Such studies demonstrated a good correlation between the participants’ self-reported signs and symptoms of periodontal disease compared to the clinical findings detected by the professional (probing depth, for example). Although Bulin et al.^26^ concluded that self-administered questionnaires would be less reliable for these specific periodontal variables, the authors reinforce and encourage the use of these tools in epidemiological studies. Therefore, bearing in mind that the OHIP 14 DP - Br aims to provide a more comprehensive measure of the discomfort, dysfunction, and disability attributed to the periodontal condition and its impact on the participant’s daily life, in the present study, the participant responded to the questionnaire about the repercussions of periodontal disease in its life. In contrast, specific periodontal parameters were not asked. The OHIP 14 DP - Br was presented as an easily understood and quick-completed instrument, which can increase cooperation in using it.

This analysis selected individuals according to the Periodontal Screening and Recording, the PSR.^18^ This index allows reproducibility, reliability, and speed in coding the periodontal conditions of individuals without the obligation to classify the intensity of clinical signs and symptoms^28^. Since the sample population of this study was divided into individuals with the presence or absence of periodontal disease, the PSR exam and its scores proved to be adequate for this purpose.

The questionnaire validated in this study showed excellent internal consistency (Cronbach’s alpha), considered above the recommended minimum level, which should be higher than 0.70.^23^ Cronbach’s alpha is one of the most widely used methods to estimate internal consistency in applied research, considering the magnitude in which the items of an instrument are correlated.^29^ In this research, Cronbach’s alpha coefficient demonstrated that the seven dimensions of the OHIP 14 DP - Br questionnaire are highly correlated and significant, with a total Cronbach’s α value = 0.945 (p < 0.001). When evaluated item by item, the internal consistency of the present study showed Cronbach’s alpha values higher than 0.9. These values are close to those presented in the original questionnaire (α = 0.928; p < 0.001).^16,30^

As in the original questionnaire, no item/question needed to be excluded, as all values exceeded 0.9, indicating good internal consistency of the instrument.^31^ Although a high Cronbach’s alpha value does not necessarily indicate a high level of consistency between the items,^32^ these high values may be related to a certain redundancy between the questions, which compromises the validity of the content, indicating that the same item can be measured something similar to another item contained in the questionnaire. This indicates the need for future research.

The reproducibility of the test-retest method’s stability is essential when assessing physiological or psychological compromises. The ICC index is a parameter used to estimate the measurement reliability induced by human response errors. Thus, an instrument with temporal stability (test-retest reliability), measurements obtained under so-called stable conditions, or even may present the same result, assuming that there were no changes in the parameters of what is being evaluated.^33,34^ The application time between the test and the retest must be long enough to avoid recall but somewhat brief to ensure that there is no change between responses to verify the temporal stability of this method. In this study, the time used was 7 to 10 days, considered appropriate for the method^35^. During this period, participants did not receive any periodontal treatment. The total ICC of the OHIP 14 DP - Br was considered excellent (ICC = 0.997, CI95%: 0.995–0.998) concerning the covered domains, producing a similar result regardless of time, environment, or examiner.^34^ Therefore, the Brazilian version can be considered a reliable and stable instrument.

The construct validity of the translated version of the OHIP 14 PD was assessed using convergent and discriminative validity. Construct validity is necessary for instruments that inquire about health completed by the individual and, in this way, guarantee that, when validated, they will evaluate the construct that these instruments intend to measure.^36^ Hypotheses are tested about the expected relationships between the PROM and other test scores using a correlation coefficient.

Convergent validity can be defined as the significant relationship between two or more measures of the same construct or related using different validation methods or instruments. A self-rating scale of the individual’s gingival health was used to explore the convergent validity with the OHIP 14 DP - Br, considering that the two instruments deal with the participants’ self-perception of oral health. Correlation coefficients indicate the strength of the association between two sets of scores, where strong correlations indicate that the scores on the two tests move in the same direction, i.e., as one increases, so does the other, and the correlation given as unfavorable, points out that the scores obtained change in opposite directions.^37^ This study’s correlation was negative and weak to reasonable strength. Hence, it can be inferred that higher scores captured by the Likert scale correlated with lower scores on the gingival health self-perception scale, with this correlation being significant for dimensions 1 (functional limitation, p = 0.046), 4 (physical disability, p = 0.021), 5 (psychological disability, p = 0.009) and 6 (social disability, p = 0.013). The highest significant negative association was demonstrated in dimension 5 (rs= -0.347; p = 0.013), which deals with the individual’s psychological incapacity. In this dimension, the questions are directed toward the consequences of periodontal disease on the patient’s mood and how they feel in front of other people because of the condition of their teeth and gum.^12^

Discriminant validity assesses or identifies differences between groups theoretically expected to verify these differences.^37^ The participants’ scores with periodontal disease (mean 30.77; SD ± 14.39) were significantly higher than those without the disease (mean 8.50; SD ± 9.50), as predicted. This difference can be interpreted as evidence for discriminant validity and indicates that the groups (with and without periodontal disease) differ. The original study also showed discriminant validity of the OHIP 14 PD questionnaire^31,16^ using other statistical methods. In both evaluated groups, the psychological incapacity domain had the highest averages. Such questions were about the feeling of sadness or shame about their teeth and gums. Masood et al.^6^ suggested that periodontal disease presents the possibility of creating a significant disadvantage due to the potential for functional limitations and adverse effects on the social and psychological relationships of that patient.

As a limitation of the present study, a notable factor is the absence of calibration of the PSR exam, given that it was routinely conducted at the UFVJM dental clinic by undergraduate students and confirmed by the professors of Periodontics. This may have reduced the internal validity of the study. Also, the minimum sample size required is highlighted, based on sampling by judgment and the non-verification of the responsiveness of the OHIP 14 DP - Br version since longitudinal data have yet to be collected. Other studies with greater recruitment of participants and using other statistical methods should be encouraged. Furthermore, as a PROM instrument, its use requires careful reflection on its intended purposes in conducting clinical studies and identifying different foci.^37^

The negative impact of periodontal disease on individuals’ quality of life has been widely studied, and evidence suggests that the disadvantage can be directly related to the severity of the disease.^9,38,39^

In addition to being clinically examined, periodontal disease should be subjectively assessed through measures that quantify its impact on quality of life.^9,40^ PROMs consist of clinical tools proficient in capturing the patient’s self-perceived health status and provide an advantageous method for clinicians to monitor changes in the health aspects of patients over time.^37^ Since the OHIP 14 DP - Br is a condition-specific PROM, it is a helpful method for analyzing the impact of periodontal disease on individuals’ daily lives.

## Conclusion

It can be concluded that the translation and cross-cultural adaptation of the OHIP14 PD questionnaire resulted in a promising PROM tool named OHIP 14 DP - Br for surveillance of the periodontal condition, with validated, stable, reliable, and consistent psychometric characteristics.

## References

[1] Haag DG, Peres KG, Balasubramanian M, Brennan DS (2017). Oral Conditions and health-related quality of life: a systematic review. J Dent Res.

[2] Churruca K, Pomare C, Ellis LA, Long JC, Henderson SB, Murphy LE (2021). Patientreported outcome measures (PROMs): a review of generic and conditionspecific measures and a discussion of trends and issues. Health Expect.

[3] Frost MH, Reeve BB, Liepa AM, Stauffer JW, Hays RD (2007). What is sufficient evidence for the reliability and validity of patientreported outcome measures?. Value Health.

[4] Nazir M, AlAnsari A, AlKhalifa K, Alhareky M, Gaffar B, Almas K (2020). Global prevalence of periodontal disease and lack of its surveillance. Scientific World Journal.

[5] Wong LB, Yap AU, Allen PF (2021). Periodontal disease and quality of life: umbrella review of systematic reviews. J Periodontal Res.

[6] Masood M, Younis LT, Masood Y, Bakri NN, Christian B (2019). Relationship of periodontal disease and domains of oral healthrelated quality of life. J Clin Periodontol.

[7] Kinane DF, Stathopoulou PG, Papapanou PN (2017). Periodontal diseases. Nat Rev Dis Primers.

[8] Ferreira MC, Pereira ACD, Almeida LSB, Martins CC, Paiva SM (2017). Impact of periodontal disease on quality of life: a systematic review. J Periodontal Res.

[9] Dahlen G, Fejerskov O, Manji F (2020). Current concepts and an alternative perspective on periodontal disease. BMC Oral Health.

[10] Slade GD (1997). Derivation and validation of a shortform oral health impact profile. Community Dent Oral Epidemiol.

[11] Pasnik Chwalik B, Konopka T (2020). Impact of periodontitis on the Oral Health Impact Profile: a systematic review and metaanalysis. Dent Med Probl.

[12] Rodríguez NI, Moral J (2017). Adaptation and content validity by expert judgment of the oral health impact profile applied to periodontal disease. J Oral Res.

[13] Guillemin F, Bombardier C, Beaton D (1993). Crosscultural adaptation of healthrelated quality of life measures: literature review and proposed guidelines. J Clin Epidemiol.

[14] Instituto Brasileiro de Geografia e Estatística, Ministry of Health. (Brazil) (2010). Brazil National Oral Health Survey.

[15] León S, Bravo-Cavicchioli D, Correa-Beltrán G, Giacaman RA (2014). Validation of the Spanish version of the Oral Health Impact Profile (OHIP 14Sp) in elderly Chileans. BMC Oral Health.

[16] Moral de la Rubia J, Rodríguez Franco NI (2017). Internal consistency and factor structure of the oral health impact profile applied to the periodontal disease in a mexican adult general sample. Univ Odontol.

[17] Raczek AE, Ware JE, Bjorner JB, Gandek B, Haley SM, Aaronson NK (1998). Comparison of Rasch and summated rating scales constructed from SF36 physical functioning items in seven countries: results from the IQOLA Project. International Quality of Life Assessment. J Clin Epidemiol.

[18] Nasi JH (1994). Background to, and implementation of, the Periodontal Screening and Recording (PSR) procedure in the USA. Int Dent J.

[19] Gagnier JJ, Lai J, Mokkink LB, Terwee CB (2021). COSMIN reporting guideline for studies on measurement properties of patientreported outcome measures. Qual Life Res.

[20] Beaton DE, Bombardier C, Guillemin F, Ferraz MB (2000). Guidelines for the process of crosscultural adaptation of selfreport measures. Spine.

[21] Keszei AP, Novak M, Streiner DL (2010). Introduction to health measurement scales. J Psychosom Res.

[22] Li L, Zeng L, Lin ZJ, Cazzell M, Liu H (2015). Tutorial on use of intraclass correlation coefficients for assessing intertest reliability and its application in functional nearinfrared spectroscopybased brain imaging. J Biomed Opt.

[23] Allen PF (2003). Assessment of oral health related quality of life. Health Qual Life Outcomes.

[24] Bland JM, Altman DG (1997). Cronbach's alpha. BMJ.

[25] Fayers PM (2000). Quality of life: assessment, analysis and interpretation.

[26] Buhlin K, Gustafsson A, Andersson K, Håkansson J, Klinge B (2002). Validity and limitations of selfreported periodontal health. Community Dent Oral Epidemiol.

[27] Gilbert AD, Nuttall NM (1999). Selfreporting of periodontal health status. Br Dent J.

[28] Landry RG, Jean M (2002). Periodontal Screening and Recording (PSR) Index: precursors, utility and limitations in a clinical setting. Int Dent J.

[29] McCrae RR, Kurtz JE, Yamagata S, Terracciano A (2011). Internal consistency, retest reliability, and their implications for personality scale validity. Pers Soc Psychol Rev.

[30] Moral de la Rubia J, RodríguezFranco NI (2017). Validation of the Oral Health Impact Profile applied to patients with periodontal disease. Rev Fac Odontol Univ Antioq.

[31] Sijtsma K (2009). On the use, the misuse, and the very limited usefulness of Cronbach's Alpha. Psychometrika.

[32] Tavakol M, Dennick R (2011). Making sense of Cronbach's alpha. Int J Med Educ.

[33] Lee KM, Lee J, Chung CY, Ahn S, Sung KH, Kim TW (2012). Pitfalls and important issues in testing reliability using intraclass correlation coefficients in orthopaedic research. Clin Orthop Surg.

[34] Koo TK, Li MY (2016). A Guideline of selecting and reporting intraclass correlation coefficients for reliability research. J Chiropr Med.

[35] Terwee CB, Bot SD, Boer MR, Windt DA, Knol DL, Dekker J (2007). Quality criteria were proposed for measurement properties of health status questionnaires. J Clin Epidemiol.

[36] Abma IL, Rovers M, van der Wees PJ (2016). Appraising convergent validity of patientreported outcome measures in systematic reviews: constructing hypotheses and interpreting outcomes. BMC Res Notes.

[37] Davidson M, Keating J (2014). Patientreported outcome measures (PROMs): how should I interpret reports of measurement properties? A practical guide for clinicians and researchers who are not biostatisticians. Br J Sports Med.

[38] Meusel DR, Ramacciato JC, Motta RH, Brito RB, Flório FM (2015). Impact of the severity of chronic periodontal disease on quality of life. J Oral Sci.

[39] Bhargava N, Jadhav A, Kumar P, Kapoor A, Mudrakola DP, Singh S (2021). Oral health related quality of life and severity of periodontal disease. J Pharm Bioallied Sci.

[40] Locker D, Allen F (2007). What do measures of 'oral healthrelated quality of life' measure?. Community Dent Oral Epidemiol.

